# Teachers’ Strategies and Technology Use for Enhancing Students’ Critical Thinking in Nursing Simulation-Based Learning: A Qualitative Pilot Study

**DOI:** 10.1177/00469580251392452

**Published:** 2025-11-11

**Authors:** Malin Forsbrand, Line Christiansen, Vicky Johnson Gatzouras

**Affiliations:** 1 Blekinge Institute of Technology, Karlskrona, Sweden

**Keywords:** critical thinking, nursing education research, simulation training, simulation-based learning, technology, qualitative research

## Abstract

In nursing education, simulation-based learning (SBL) is often used to bridge theoretical knowledge and practical application, supporting nursing students in developing their critical thinking (CT) skills. Despite the benefits of using SBL in nursing education, research gaps remain in understanding student learning outcomes. Furthermore, there is a lack of studies describing the specific use of technology in its application. The objective of this study was to explore strategies for learning and the use of technology to enhance nursing students’ CT within the SBL context. This research was conducted as a qualitative pilot study, using a semistructured interview technique to gather insights from teachers at 2 universities in the south of Sweden. The obtained data were analysed in accordance with the phenomenographic analysis introduced by Sjöström and Dahlgren. The results revealed participants’ perceptions of useful strategies for student learning and different ways of using technology. In particular, the results are reflected in 5 descriptions of categories: *motivating environment, facilitating preparations, active participation, student-centeredness* and *reflective observations*. While the findings may not be directly applicable to clinical practice, the study’s findings offer examples of effective strategies for student learning and technology use, thus providing valuable guidance for teachers implementing SBL in nursing education. To gain a more comprehensive understanding of using SBL as a teaching method, future research should aim to investigate nursing students’ experiences of how CT is promoted via simulations.

**What do we already know about this topic?**

Simulation-based learning is often used in higher education to bridge theoretical knowledge and practical application to support students in developing their critical thinking skills, but gaps remain in understanding student learning outcomes and use of technology in its application.

**How does your research contribute to the field?**

This study explores teachers’ perspectives on effective strategies and technology integration to enhance critical thinking in SBL.

**What are your research’s implications toward theory, practice, or policy?**

The findings offer practical guidance for teachers in nursing education to design effective SBL approaches and inform future pedagogical development.

## Introduction

Active learning methods are often used in higher education to support students in the practical implementation of theoretical knowledge and in the development of their critical thinking (CT) skills.^1-3^ This approach is especially important for nursing students who are required to integrate theory and practice to ensure the purposeful and safe healthcare of patients in their future professional practice.^4^ Nevertheless, integration is often perceived as difficult by nursing students and thereby discussed as an ongoing educational challenge.^5^

Educational simulation, also known as simulation-based learning (SBL), is often used as an active learning method in nursing education. SBL has been recognised as an effective learning method, as it enables students to work in an environment that closely resembles that of a healthcare setting.^4,6^ Through simulation, a study attempted to replace real patients with virtual standardised patients or technologies and methods capable of reproducing actual clinical scenarios for educational purposes.^7^ In this way, SBL can be considered an active learning method in which technological tools can play a significant role in supporting nursing students in the application of theoretical knowledge to practical situations and in the development of their critical thinking (CT) skills. However, certain gaps remain in the science of simulation, particularly in the aspect of student learning outcomes, which have been identified as one of the greatest gaps in this field.^4,8^ In addition, there is a lack of studies describing how technologies are used in SBL to promote students’ learning.^6^

As teachers within higher education have a key role in supporting students in their learning process, the abovementioned challenges must be addressed. In particular, researchers must investigate which strategies are considered effective for student learning and how technologies are used within SBL to support nursing students and enhance their learning outcomes, specifically their CT. Such research can contribute important knowledge for bridging the gap concerning CT in simulation research and can provide practical examples of how technology can be used to promote students’ learning.

## Review of Literature

### Active Learning in Nursing Education

Within nursing education, the basic pedagogical principle is built on theoretical and practical knowledge, along with valuable philosophical questions that are critical to nurses’ professional functions.^5^ In this field, theoretical knowledge is mainly focused on nursing theories, pathophysiology and educational theories, while practical knowledge is based on experiences and, to some extent, tacit knowledge or familiarity. For nursing students, developing the ability to integrate theory and practice is necessary to ensure purposeful and safe healthcare for patients; however, it is still discussed as an educational challenge.^4,5^ Previous studies^9,10^ have described the lack of coordination between theory and practice as one of the main problems within nursing education, specifically the difficulties encountered by nursing students in the practical implementation of theoretical knowledge.

As teachers within higher education have a key role in supporting students in their learning process, strategies for student learning must be built on effective learning methods. One such method is the use of simulation, in which the goal is to activate nursing students’ own learning in terms of problem solving and critical and holistic thinking based on an environment closely resembling that of a healthcare setting.^7^

### Simulation and Simulation-Based Learning

Simulation has emerged as an essential component of health and medical education and training; thus, it has become more commonplace throughout the healthcare system in recent years.^11,12^ ‘Simulation’ can be defined as a technique ‘to replace or amplify real experiences with guided experiences that evoke or replicate substantial aspects of the real world in a fully interactive manner’.^13^ Thus, it is about imitating something from the ‘real world’ that participants can experience, although such experiences can be controlled in different ways. Different types of simulations, such as tabletop (e.g., group discussions or computers) and technical (e.g., patient mannequins) simulations, have been described. In nontechnical simulations, the scenario is the focus and role-playing can be included.^14^

Simulation is often an integral part of educational programmes in different areas within higher education, such as educating and training healthcare professionals,^12^ police officers^14^ and naval officers.^15^ Educational simulation, known as SBL, is an active learner-centred method, in which the educator acts as a facilitator of learning.^16^ SBL is defined as ‘a dynamic process involving the creation of a hypothetical opportunity that incorporates an authentic representation of reality, facilitates active student engagement, and integrates the complexities of practical and theoretical learning with opportunity for repetition, feedback, evaluation, and reflection’.^17^ SBL can be performed either in a clinical setting, using ‘in situ’ or ‘on-site’ simulation or in a simulation centre.^18^ In this way, SBL is a pedagogical active learning method that facilitates learning in a safe environment with the opportunity to gain experience and practice without the risk of harming actual patients.^19^

Despite the benefits of using SBL as a teaching method within nursing education, certain gaps remain in the science of simulation, with researchers identifying rigour and student learning outcomes as the greatest gaps.^4,8^ Thus, exploring how technology is used in SBL to support nursing students’ learning outcomes is one of the main motivations of the current study.

### Simulation-Based Learning Supported by Technology

To date, an expanding area of technological solutions has emerged to support SBL in nursing education.^20^ Studies have reported that the use of technology in SBL can ensure equitable learning opportunities by providing the same content and learning environment for all students, making it a resource-efficient option due to its low costs.^21-23^ However, due to the wide range of technologies used in simulation, no clear definition of simulation technology in higher education domains has been proposed.^24^

In nursing education, there are different types of simulation technologies, including high- and low-fidelity mannequins, partial task simulators and virtual reality (VR),^7^ which use computers and standardised patients to create realistic learning and evaluation settings. In addition, various forms of simulation games in nursing education have emerged and have been reported to have the potential to stimulate CT.^22,25^ More commonly known technologies, such as smartphone/tablet applications for feedback and web meeting tools for virtual meetings, can stimulate dialogues between students and teachers, adjusting the former’s learning focus and ensuring the latter’s accurate assessment of learning outcomes.^26^ However, only a few studies have described how technologies are used in SBL to promote students’ learning.^6^ The present study explores the types of simulation technologies described above in terms of how they are used to support students in their learning processes and their development of CT.

### Critical Thinking as a Learning Outcome

The learning outcome of interest in this study is students’ CT. Over the years, this concept has been subject to much detailed scholarly work and has often been regarded as one of the most important outcomes expected of university graduates.^27^ In 1990, the American Philosophical Association defined ‘critical thinking’ as ‘a judgement which is purposeful and self-regulatory and results in a process of interpretation, analysis, evaluation, and inference, as well as explanation of the evidential, conceptual, methodological, criteriological, or contextual considerations upon which that judgement is based’.^28^ All these meanings can be traced more or less directly to different theoretical perspectives on CT based on previous and more recent traditions from the history of ideas.^29^

To clarify where the content-related focal point lies from these perspectives, the authors of the current study chose to study CT from a holistic perspective based on Barnett’s^30^ theoretical framework on CT in higher education. Developing CT skills in nursing students has been found to be critical for achieving success in nursing education and has been described as a positive attribute for future professional practice.^31^ Hence, the objective of the present study was to explore strategies for learning and the use of technology to enhance nursing students’ CT in the SBL context.

## Methods

### Design

**Figure 1.**
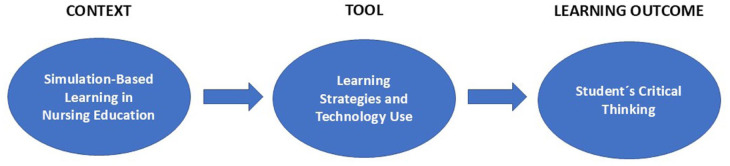
Conceptualisation of the study.

A qualitative method with an exploratory design was used to explore strategies for learning and the use of technology to enhance nursing students’ CT in the SBL context. This research was designed as a pilot study within a course in higher education pedagogy at Blekinge Institute of Technology, Sweden. Furthermore, an inductive approach was used to explore the data, indicating that the study explored a theoretical direction from the data rather than conducted a process of theory testing.^32^ The conceptualisation of the study is based on the context of SBL in nursing education, in which strategies for learning and technologies are used as tools to attain the learning outcome of developing students’ CT (Figure 1). The research questions (RQs) were as follows: (1) What types of strategies for learning are considered effective to enhance nursing students’ CT and (2) How are technologies used in SBL? The Standards for Reporting Qualitative Research (SRQR) were used to improve the transparency and clarity of the outline of this study.^33^

### Sample

In this study, 3 participants, 2 males and 1 female, aged 38 to 43 years were included. The participants were employed as higher education teachers at the nursing programmes in 2 universities, namely Blekinge Institute of Technology and Kristianstad University, in southern Sweden. The participants had 1 to 3 years of pedagogical experience with simulation training, and their academic backgrounds included positions as adjunct and associate professors. A purposive sampling strategy regarding experience of SBL and level of academic degree was used to capture variations of strategies employed in facilitating learning and the use of technology related to SBL. The main inclusion criterion involved participants’ experience of working with SBL, in which their own perceptions of the experience of relevance for this study determined participation. Due to the timeframe of this study, nonproficiency in the Swedish language was set as an exclusion criterion to facilitate the analysis process.

To recruit presumptive participants, the department heads responsible at the respective universities of interest were asked for permission before sending the study invitations via e-mail. Due to no respondence, a reminder e-mail was sent out. Eventually, 3 presumptive participants replied to the invitation and expressed interest in participating. They were then sent additional information about the study’s background, objective and approach, along with an informed consent, which they were asked to sign.

### Data Collection

Individual semistructured interviews were conducted digitally via Zoom, with audio-recording, during the spring of 2023. To ensure the clarity of the questions included in the interview guide, to estimate the time length of the interviews and to train the authors in the use of appropriate interview techniques, a pilot interview was conducted in advance with a colleague working as a teacher at the Department of Health, Blekinge Institute of Technology, Sweden. The questions were then adjusted accordingly. This pilot interview was excluded from the analysis.

The interviews were performed independently by either 1 of the 2 main authors to limit any pre-understandings regarding experience of teaching in SBL. This procedure was chosen due to the authors’ different teaching backgrounds where 1 author had previous experience of teaching in SBL in nursing education. Further, an interview guide was used. The questions were designed to elicit information related to CT and technology use by exploring the participants’ experiences and perceptions (Appendix A). Using an interview guide allowed the authors to collect data in a uniform way while maintaining focus on the study area of interest. The participants’ initial responses were followed up with prompt questions to elicit more detailed information and to support data saturation, such as asking them to clarify their statements or provide practical examples.

### Data Analysis

The obtained data were analysed using a phenomenographic approach, in accordance with the revised guidelines proposed by Sjöström and Dahlgren^34^ and originally developed by Dahlgren and Fallsberg.^35^ Although this study was not designed as a phenomenographic study per se, using this approach allowed the authors to identify qualitative data regarding the participants’ different experiences and understandings based on a collective human perception.^32^

Next, to interpret meanings, generate depictions of the research setting and identify patterns, the analysis was conducted in consecutive steps based on a constant interplay between the steps. In the first step, each interview was transcribed verbatim and read individually by each author to help them become familiar with the content. The following steps were carried out conjointly: the identified significant strategies related to the aim were summarised and organised into preliminary categories based on their differences and similarities. These categories were subsequently compared to establish clearly defined boundaries between the strategies and the ways in which technology was used within each category. The resulting categories were then named to emphasise their essence (see example below). The analysis resulted in 5 categories of description. In the final step, a contrastive comparison was made to determine the unique characteristics of each category.

Example: ‘It is valuable with technical features to mimic reality as much as possible’ (significant element); Lifelike (preliminary category); Realistic setting (subcategory); Motivating environment (category of description).

## Ethics

This study was conducted in compliance with the ethical guidelines outlined in the Declaration of Helsinki.^36^ An ethical statement by the regional ethics review board in southeast Sweden was obtained (Dnr: 875-2023). Prior to participating in the interviews, written informed consent was obtained from the interviewees. Additionally, verbal consent was recorded at the beginning of each interview.

## Results

The results of this study showed the participants’ perceptions of useful strategies for student learning and the different ways of using technology to enhance students’ CT in the SBL context. The final categories of description and its subcategories are presented in Table 1. The results are described below, along with the coded citations.

**Table 1.** Overview of the Categories of Description, along with their Subcategories.

Categories of Description	Subcategories
Motivating environment	Good teaching climate
	Realistic setting
Facilitating preparations	Theoretical preparations
	Practical preparations
Active participation	Student interaction
	Modification
Student-centeredness	Feedback
	Situation-driven approach
Reflective observation	Monitoring
	Debriefing

### Motivating Environment

This category includes descriptions of establishing a good teaching climate and realistic settings to create motivating learning environments, which were perceived by the participants as enhancing students’ CT. In particular, the participants expressed that, in simulation environments, the teacher had the possibility of adapting scenarios to mimic reality. Their role as teachers was to talk, provide support and encourage the student group during simulations. To maintain focus on reality during simulations, the teacher also provided students with practical examples based on their own clinical experiences with real-life patients. Making the students feel stressed was described as nonbeneficial for the development of students’ CT. Statements indicating that simulations should be fun and exciting, rather than stressful learning opportunities, were emphasised, as in the following example:

You learn most when it’s exciting and fun, not stressful (B)

The use of simulation technologies was described as a central part of creating a motivating environment, because they offer the possibility of establishing a setting closely resembling that of a healthcare setting. The value of having high-fidelity technologies to imitate reality is duly recognised, and the participants perceived how the simulations became more alive with the use of such technologies. The participants used high-fidelity mannequins (e.g., a digitally connected simulation doll) and more common technologies (e.g., tablet device, microphones, cameras and large screens) to monitor, communicate and modify simulations. Furthermore, communication platforms, such as Zoom, were used to simulate scenarios and train students in conducting distance health dialogue with patients, as commonly practiced in healthcare settings.

### Facilitating Preparations

This category includes descriptions of facilitating preparations, in terms of theoretical and practical preparations, which were perceived to enhance students’ CT. Pre-simulation lessons were applied to prepare the students theoretically prior to the simulations. The students were offered a lecture the day before and were expected to have read about the subject in advance to ensure maximum preparation for the simulations. To further support their theoretical preparation, students engaged in group discussions of various patient cases prior to the practical sessions. In connection with the simulations, the students received a brief review of actual patient cases.

Before simulation, we have a small briefing together, where I tell them about the case and what they will encounter (B)

Furthermore, the teacher must be prepared theoretically and practically before simulations to alternate theory and practice during the sessions, thereby supporting the development of the students’ CT. In terms of theoretical preparation, the participants expressed a preference for teaching ‘by the book’ and preparing by reviewing course literature and evidence-based material. In terms of practical preparation, the participants conducted functional tests of the technical equipment to ensure it operated correctly and configured it according to the specific patient case for each scenario. This practical preparation was a prerequisite for using technology effectively and for achieving a high level of realism in the simulated scenarios in order to facilitate the development of students’ CT.

### Active Participation

This category contained descriptions of active participation through student interactions and modifications of scenarios with simulation technologies, which were perceived to enhance students’ CT. The learning process was initiated through activity and collaborative interactions between students, in which they helped one another solve a patient case in each scenario. This was described in terms of problem- and peer-based learning, wherein variations of peer-learning constellations occurred. The participants described how the group dynamics emboldened the students to act accordingly during simulations.

During the simulation, the available technologies allowed the teachers to observe and modify the scenarios. These included a high-fidelity mannequin that was used as a fictive patient and a tablet device. These simulation technologies made it possible for teachers to remotely control and adjust the functions of the mannequin, while the students checked and monitored the fictive patient’s vital signs, such as pulse and blood pressure. In addition, technologies were used to communicate as real-life patients and to give instructions to students. Furthermore, other common technologies, such as microphones, were placed inside the control and simulation room and were used to facilitate communication. Overall, the use of simulation technologies enabled dynamic scenario outcomes, encouraging students to engage in critical thinking and develop solutions that promoted high-quality patient care.

### Student-centeredness

This category includes descriptions of feedback and situation-driven approaches perceived to enhance students’ CT. The participants expressed that the feedback and teaching approaches applied during simulations must be adapted to students’ needs. In particular, the feedback provided during the simulations was tailored to each student’s ability of applying theoretical knowledge into practical actions, fostering a more individualised learning experience. To identify individual differences in knowledge, follow-up and/or in-depth questions were employed to challenge the students and stimulate deeper reflection.

If there are two students and one of them is more active than the other one, you need to adapt so that everyone gets the opportunity to take part in the simulation (C)

Furthermore, feedback was given during and after simulation to motivate, clarify and explain, thereby enhancing student-centredness. The participants described that a balance between the amount of feedback given during simulations was required so as not to affect students’ performance. The mediation of deficiencies was usually raised afterwards. This strategy was considered helpful in developing students’ CT.

To use a situation-driven approach, the participants emphasised the importance of being flexible and responsive as teachers. They stated that some students may need to be challenged more, while other students need more time to deal with the situation. Although the simulations had a basic structure, student-centredness had to be considered, depending on which students were participating in the learning situation. In cases where the teachers discovered weaknesses in their students’ skills and abilities, they set aside time to allow these students to continue simulations individually. This strategy was described by the participants as useful in assessing individual skills.

### Reflective Observation

This category included descriptions of using simulation technology for monitoring and debriefing, which were perceived to improve students’ CT. To monitor the simulations, microphones, cameras and large screens were used, allowing other students to observe the simulations remotely. The cameras were used to capture different angles from the simulation room, which were then shown on large screens so that both the teachers and students outside the simulation room could observe how the students acted during the scenarios. Using microphones also made it possible to hear how the students communicated with each other and with the fictitious patient. Such technology brought a great deal of freedom and opportunity for the teachers to control the situation and pay attention to small things while ensuring the smooth imitation of reality. Furthermore, the use of technologies allowed a larger number of students to be involved by reflectively observing the scenarios. This, in turn, made it possible for all students to have a subsequent debriefing about how the scenario progressed.

The most important thing about simulation is that it provides a dimension of real time (C)

Having a debriefing after each simulation was highlighted as central to the development of students’ CT. During the debriefing session, the students were encouraged to reflect on and discuss the progression of the scenario, focusing on both their successful actions and areas for improvement. This reflective session was regarded as an effective strategy for the students to reflect on their current knowledge.

It becomes an entirety when linking theory and practice after the simulation (A)

## Discussion

### Principal Results

The principal findings of this study include *motivating environment, active participation* and *reflective observation*, all of which can be linked to theoretical foundations shown to enhance students’ CT. The simulation technologies were used in 3 different ways: (1) to create realistic setting, (2) to modify the scenarios and (3) to monitor the simulations.

The findings of this study indicate that fostering a *motivating environment* through the use of simulation technologies was perceived as beneficial for nursing students’ practical implementation of their theoretical knowledge. Furthermore, providing a good teaching climate facilitated students’ learning and enhanced their CT skills development by increasing their learning motivation. By demonstrating interest in students, particularly in their learning, prior knowledge, experiences and backgrounds teachers can establish a foundation for a positive and supporting teaching climate.^37^

Furthermore, given that learning is largely based on unique conditions and abilities, teaching must constantly be conducted in dynamic collaborations with student groups and the unique individuals who comprise them.^37^ Thus, as highlighted by the participants, teacher flexibility and responsiveness, are essential for ensuring that dynamic interactions function effectively. However, the results cannot offer a clear explanation of whether providing a motivating environment can guarantee the in-depth development of nursing students’ CT. This is mainly because several factors interacting with each other can affect students’ learning outcomes.^5^ Thus, more strategies for learning must be considered when evaluating CT in a simulation setting.

Additionally, *active participation* was emphasised as a significant learning strategy for nursing students, whereby CT was enhanced through interactive student engagement and scenario adaptations facilitated by simulation technologies. Notably, the technology-related findings of this study are dependent on universities’ access to simulation technologies. Thus, the conditions for nursing simulations will differ between universities. However, previous research suggests that the type of technology used in simulation does not necessarily influence learning outcomes, indicating that both high- and low-fidelity simulations can be equally effective in facilitating student learning.^38^ As this has not yet been thoroughly proven,^24^ this could be an interesting topic for future research.

A comparison between the findings of CT as a learning outcome in this study and Barnett’s^30^ framework of CT in higher education yields interesting insights. In accordance with Barnett’s framework, criticality can occur in 3 forms: critical reasoning, critical self-reflection and critical action, which can be released on 4 levels ranging from simpler forms to more advanced ones: (1) critical skills, (2) reflexivity, (3) refashioning of traditions and (4) transformatory critique. Based on this notion, problem solving in practice, as demonstrated by the findings of this study, is related to the first level of critical skills, which deals with analysis and evaluation in the form of critical action. This is the lowest level of CT. However, as further indicated by this study, *reflective observation* through monitoring and debriefing was described as central to enhancing nursing students’ CT. Thus, in relation to Barnett’s framework, these findings correspond to the second level of reflexivity, which encompasses critical reflection on one’s own understanding of disciplinary norms, self-reflection on personal ambitions, and reflective practice through self-directed learning. This means that the *reflective observation* that occurred during and after the simulation accounted for criticality via critical reasoning, critical self-reflection and critical action. Thus, when combined, the 2 lower levels of criticality were activated using SBL. This is a noteworthy finding because it implies that SBL cannot support nursing students in terms of developing the highest level of criticality, which involves criticism of the discipline’s knowledge framework, ethical responsibility and development as a person. From a broader perspective, all these aspects are important in improving healthcare and ensuring that accurate and safe patient care is provided.

Overall, the use of SBL as an active learning method in nursing education cannot be used as a single method to achieve all levels of CT that are necessary for prospective nurses to ensure purposeful and safe care of patients in professional practice.^4^

## Methodological Considerations

A demonstration of rigour or quality in the conduct of a study is essential in all research. In the present study, quality aspects are discussed in the criteria of trustworthiness outlined by Lincoln and Guba.^39^ These criteria are often referred to as ‘standards of trustworthiness to qualitative research’ and consist of 4 concepts: *dependability, credibility, transferability* and *confirmability.*

The use of a semistructured interview guide in this study demonstrates *dependability.* The interview guide ensured that the authors collected data similarly, especially because the interviews were conducted separately, with only 1 of the 2 authors participating in each interview. One limitation of this study is that the interview guide was not formally validated. However, it was pilot-tested in advance with a teacher experienced in simulation training for nursing students, which helped ensure its clarity and relevance.

Furthermore, in terms of *credibility*, 3 main aspects of the present study contribute to strengthening its quality. First, a varied sample selection concerning the level of academic degree was deliberately recruited to gather participants who contributed with different ways of understanding the outcome of interest. Second, triangulation was used, which meant that all steps in the analysis were performed jointly by the 2 main authors. During this process, the various stages were carefully documented, and the authors held repeated discussions to ensure confidence in the ‘truth value’ of the data and its interpretation. Finally, the description of the results was written together and reviewed by the third author, to minimise any subjective influence from the individual authors’ pre-understanding of the context. To heighten the transparency of the interpretation process, the authors used several quotes in the final presentation of the results. However, the small amount of data limited the scope for fully exploring the full range of variations in participants’ experiences (similarities and differences), as is typical in a complete phenomenographic study. Consequently, the analysis focused on identifying indicative patterns of meaning, providing an initial mapping of potential categories of description and possible variations. As a result, the depth of understanding variations is limited, and the findings should be interpreted as exploratory.

Finally, *transferability* can be seen as a generalisation through the recognition of patterns, as the results align with well-recognised patterns within the pedagogical literature of higher education.^36^ Based on this perspective, the results of this pilot study can serve as a foundation for future research. It should be noted that the small sample size, consisting of only 3 teachers from 2 universities in the south of Sweden, might limit the transferability of the findings to implement in other nursing education programmes with different curricula. Although data saturation was achieved in each interview, a larger sample size would have generated more comprehensive data, thereby increasing the transferability of the study’s findings. To strengthen the *confirmability* of this study’s findings, the authors recommend full-scale investigations, including the selection of similar cohorts of participants from several universities and the use of the same study purpose.

## Implications for Practice

As with most phenomenographic studies,^34^ the results of the present study cannot be applied directly to clinical practice. Nevertheless, the present study offers examples of effective strategies for student learning and technology use. Simultaneously the study provides valuable guidance for teachers striving to use SBL as a method to facilitate the implementation of theoretical knowledge into practice, thus enhancing nursing students’ CT as a learning outcome. It would be recommended that these strategies are introduced in the early stages of nursing education and consistently applied throughout the educational programme, thereby supporting the ongoing development of nursing students CT.

## Conclusions

The results of this study show variations in participants’ perceptions of useful strategies for student learning and different ways of using simulation technologies. These findings can, to some extent, be linked to the theoretical foundations shown to enhance students’ CT. When using SBL as a learning method in nursing education, the findings indicate that, in accordance with Barnett’s framework, only the 2 lowest levels of CT can be promoted. In this study, we focused on technology use and strategies for learning. To gain a more comprehensive understanding of using SBL as a method, future research should aim to investigate nursing students’ experiences of how CT is promoted via simulations.

## Supplemental Material

sj-docx-1-inq-10.1177_00469580251392452 – Supplemental material for Teachers’ Strategies and Technology Use for Enhancing Students’ Critical Thinking in Nursing Simulation-Based Learning: A Qualitative Pilot Study Supplemental material, sj-docx-1-inq-10.1177_00469580251392452 for Teachers’ Strategies and Technology Use for Enhancing Students’ Critical Thinking in Nursing Simulation-Based Learning: A Qualitative Pilot Study by Malin Forsbrand, Line Christiansen and Vicky Johnson Gatzouras in INQUIRY: The Journal of Health Care Organization, Provision, and Financing
