# Supplementary material for: Teachers’ Strategies and Technology Use for Enhancing Students’ Critical Thinking in Nursing Simulation-Based Learning: A Qualitative Pilot Study
Source: Inquiry. 2025 Nov 11;62:00469580251392452. doi: 10.1177/00469580251392452 (PMC12605884; doi:10.1177/00469580251392452)
Supplement: sj-docx-1-inq-10.1177_00469580251392452 – Supplemental material for Teachers’ Strategies and Technology Use for Enhancing Students’ Critical Thinking in Nursing Simulation-Based Learning: A Qualitative Pilot Study [file sj-docx-1-inq-10.1177_00469580251392452.docx]

**Appendix A**

*Semistructured Interview Guide*

1. Questions about background characteristics: age, gender, employment, educational

background, academic degree and pedagogical experience.

2. Can you tell me about your experience using simulation-based learning ( SBL )

as a learning method?

3. Have you been using any kind of technology during simulations?

4. If technology was used, what type of technology was it, and how did you use it?

5. How do you, as a teacher, act to support the development of students’ critical

thinking during simulations? (Do you have any specific strategies?)

6. From a pedagogical perspective, how do you perceive your role as a teacher during

simulations?

7. Do you have any additional thoughts of relevance for SBL that you would like

to discuss/share?
